# Case Report: Diabetic urinary auto-brewery and review of literature

**DOI:** 10.12688/f1000research.52461.1

**Published:** 2021-05-20

**Authors:** Abdulrahman A. Alduraywish

**Affiliations:** 1 Department of Internal Medicine, College of Medicine, Jouf University, 2014 Sakaka, Al-Jouf, 42421, Saudi Arabia

**Keywords:** Urinary auto-brewery, Candiduria, Blood ethanol, Amphotericin B, Type 2 diabetes mellitus

## Abstract

**Background:** Although candiduria is an expected encounter and should not be surprising in uncontrolled diabetes with glucose-enriched urine, urinary auto-brewery is rarely thought of by diabetologists. Moreover, endogenous ethanol production in humans from gut microbiome, urinary tract fungi and bacteria, and intermediary metabolism, has been reported for a long time, particularly in diabetics.

**Case description:** To alert physicians to the overlooked implication of endogenously produced ethanol both as a biomarker for poor control of diabetes and as a complicating factor, we report this case of an elderly male smoker alcohol-abstinent insulin-dependent Type 2 diabetic patient. Because of circumstantial treatment and incompliance for one week, he developed endogenously produced alcohol intoxication. We proposed candidal urinary auto-brewery evidence sourced from the case history, urinalysis, and culture/identification tests - without excluding other sources. Fortunately, his diet and glycemic control were fairly controlled and, liver and kidney functions were almost normal. Amphotericin B I/V for five days, insulin, and a fluid therapy regimen greatly improved the case and cleared both the candiduria and ethanol from the urine and blood and the patient regained his base-line normal life.

**Conclusion: **Symptoms of alcohol intoxication should be expected in patients with uncontrolled diabetes that most often correlates with candiduria and/or constipation. These symptoms can be exaggerated in those already suffering a degree of dementia and/or comorbid psychiatric/neurologic affections. Direct wet mount examination of urine under phase contrast microscopy would show the budding yeast cells.  Appropriate antifungal, insulin and fluid therapies regained the base-line norms.

## Introduction

Auto-brewery syndrome, in the setting of little alcohol consumption or teetotalers, is rarely reported probably because of under diagnosis. ^1, 2^ High endogenous ethanol production and blood levels, due mainly to a sedentary lifestyle with a high carbohydrate diet and enhanced gut fermentation, is documented in the literature as early as the 1950s. ^3^ Altered microbiome, caused by diet changes, immune dysfunction and other diseases and medications, is a major contributor. Such dybiogenesis is implicated in worsening and/or causation of systemic low-grade inflammation, metabolic endotoxemia, autoimmunity, changes in microbiotal metabolite/enteroendocrine hormonal profile, hyperglycemia, fatty liver, and an expanding list of cardiovascular, gastrointestinal, neurologic, oncologic, metabolic, respiratory and psychiatric disorders. ^4, 5^ Of concern, *Candida albicans* is able to produce 1 mg/hr of ethanol per gram of intestinal content. ^6^ Considering the gut as the source, a minute amount of endogenous ethanol is produced as part of normal digestion. However, significant draft in the microbiome profile towards fermenting yeast (most commonly *Saccharomyces* and *Candida* species) and bacteria as pathogens, particularly in patients with co-morbidities, e.g. diabetes mellitus (DM), inherited and acquired reduced liver efficiency and the immune-compromisation with/without underlying gut problems, accumulates measurable alcohol levels in blood and breathe. ^1- 3, 7- 9^ After ingesting a carbohydrate-rich meal, a healthy subject had a measurable level of blood ethanol (physiological-blood ethanol; 0.3 ± 0.41 mg/dL) this becomes 16-folds higher in type 2 DM (T2DM) patients (4.85 ± 3.96 mg/dL). The latter showed levels significantly higher than liver cirrhosis patients (3.45 ± 2.65 mg/dL) that indicate that increased production and/or reduced clearance could be implicated - as proven by a more than 36-fold increase in patients with both DM and liver cirrhosis (10.88 ± 5.36 mg/dL). However, levels did not reach intoxication. ^8^

Most of these studies did not appreciate the other sources of endogenous ethanol; urinary fermentation particularly in the milieu of diabetic hyperglycemic/dyslipidemic/ketotic intermediary metabolism and glycosuria. The known susceptibility of these patients to carriage/infection with the sugar-fermenting *Candida* species is usually overlooked. ^10- 13^ The present case report is a red flag for diabetologists to start appreciating urinary auto-brewery among diabetic patients as a potential complicating factor.

## Case presentation

An 85-year-old diabetic male patient, who never drank alcohol, was diagnosed with T2DM 20 years ago; he was managed with diet control and oral hypoglycemics. However, he was shifted to insulin therapy over the last five years to improve glycemic control. He had a long history of renal stone formation. His family gave a history of epilepsy for the past three years, and he was given Levetiracetam 1000 mg for six months that was reduced to 500 mg for six more months. He was then maintained on Gabapentin 400 mg twice daily until now. He is a mild smoker. In the last three years, he experienced marked constipation that could last a few days. In addition, he was always on multi-vitamin, multi-mineral and Cod-liver oil food supplements.

One week before admission, due to his wife’s sickness (she took care of him) his condition worsened due to a change in diet and therapy incompliance. Initially, he started to experience dizziness and an altered mental state. Then, his condition gradually deteriorated and he had urinary and fecal incontinence, agitation, insulting and threatening others, refusing help, and refusing to shower and change his spoiled clothes. However, they did not give a history of seizure attacks during that period.

After one week, his wife recovered and started to take care of him. She noticed that his urine was milky and his mental state significantly deteriorated. On November 2, 2019, the family transferred him to El-Rehab-2 Private Hospital (Assiut, Egypt), where he was admitted to the Department of Internal Medicine. On examination at admission, he was confused, disoriented to time, place and person, dizzy and dehydrated. His vital signs were stable, his weight was 60 kg and height was 170 cm, but no recent weight loss was revealed from his clinical history. Abdominal examination showed mild tenderness over the supra-pubic area, the chest and heart examination was unremarkable. Abdominal ultrasonography of the kidneys, urinary tract and liver was unremarkable. A complete laboratory work up was done including blood and urine ethanol, urinalysis, urine culture and sensitivity. The major finding was hyperglycemia with fair glycemic control as indicated from HbA1c ≤7.6%, high blood and urinary ethanol content (measured by enzymatic colorimetric kit, cat#TBS2090; Tribioscience, Palo Alto, CA, USA – lower detection limit of 0.1 mg/dL). Complete blood count revealed mild normocytic-normochromic anemia with relative/absolute monocytosis ( Table 1). The mid-stream fresh sample of urine was cloudy, positive for ethanol (= 580 mg/dL), acidic (pH 5.5), negative for proteins, highly positive for glucose (+++), and mildly positive for ketones (+) and lactate (+) tested by urinary dip strips. The urine showed pus cells (55/HPF) and a few red blood cells (5/HPF). Numerous budding large yeast cells (nearly 1/4 of pus cell size) were easily identified upon direct examination of a wet mount of the sample sediment between slide and cover on a phase contrast microscope.

**Table 1.** The changes in blood ethanol and laboratory workup of the case before and five days after treatment. Data shown are contents of the investigated parameters and their normal reference range/cut-off values. CV = coefficient of variation from the mean, and, SD = standard deviation from the mean.

Parameter		Before	After	Range/cut-off
Ethanol	Plasma, mg/dL	110	undetectable	<10 mg/dL
Serum Chemistry	Sodium, mM/L	146	147	135-150
	Potassium, mM/L - Low	3.4	4.1	3.5-5.5
	Chloride, mM/L	105.5	103.5	96-108
	Bicarbonate, mM/L	23.6	24.1	22-25
	Urea, mg/dL	24	21	15-45
	Serum Creatinine, mg/dL	1.0	0.9	0.7-1.4
	Total bilirubin, mg/dL	0.8	0.82	0.5-1.2
	Direct bilirubin, mg/dL	0.21	0.20	≤0.25
	Indirect bilirubin, mg/dL	0.55	0.57	≤1.0
	Aspartate Transaminase (AST), U/L	38	36	≤40
	Alanine Transaminase (ALT), U/L	41	40	≤45
	AST/ALT ratio	0.93	0.90	<1
	Alkaline Phosphatase, U/L	134	110	44-147
	Total proteins, g/dL	6.71	6.95	6.6-8.1
	Serum albumin (A), g/dL	3.5	3.8	3.3-5.2
	Serum globulin (G), g/dL	2.81	2.55	2.0-3.5
	A/G ratio	1.25	1.49	1.1-1.5
	Fasting blood glucose, mg/dL	190	98	70-110
	Post-prandial blood glucose, mg/dL	295	132	≤140
	Hemoglobin A1c, %	7.25	7.10	˂6.4 (Fairly control ≤7.6)
Complete Blood Count	Hemoglobin (Hb), mg/dL - Low	11.5	11.7	12.6-17.4
	Red Blood Cell count, 10 ^6^/μL	4.32	4.41	3.8-5.8
	Haematocrit, %	38.6	39.7	37-51
	Mean Corpuscular Volume, fL	89.5	93.7	80-100
	Mean Corpuscular Hb, pg	27.7	30.2	27.4-34
	Mean Corpuscular Hb Concentration, g/dL	31	33	31-36
	Red Cell Distribution Width-CV	14.9	15.3	12-16
	Red Cell Distribution Width-SD	49.4	44.1	35-55
	White Blood Cell count, 10 ^3^/μL	9.3	8.4	4.5-11
	Lymphocytes, 10 ^3^/μL (%)	2.9 (31)	2.78 (33)	0.8-4.8 (18-44)
	Monocytes, 10 ^3^/μL (%) - High	1.1 (12.4)	0.8 (9.5)	0.2-0.9 (0.0-10)
	Neutrophils, 10 ^3^/μL (%)	5.3 (56.6)	5.1 (60.7)	5-7.7 (35-80)
	Platelets, 10 ^3^/μL - aggregated will	255	318	150-440
	Mean Platelet Volume, fL	9.4	10.9	8-12
	Plateletcrit, %	0.239	0.317	0.1-0.5
	Platelet Distribution Width-CV, %	15.9	14.3	8-18

**Figure 1.**
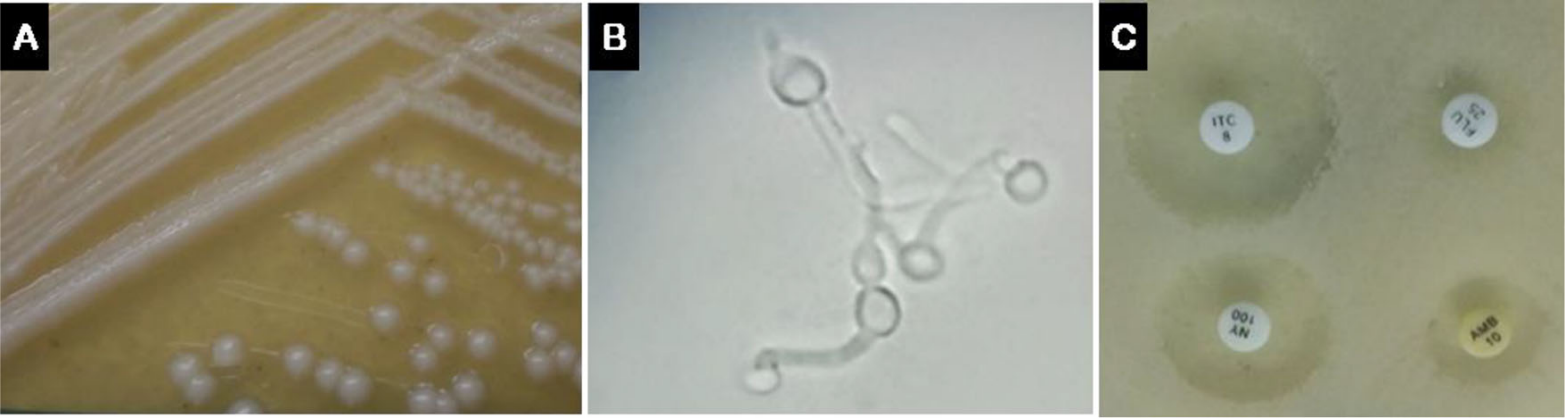
A) Typical colony characteristics of *Candida* sp *.* on Sabouraud Dextrose Agar. B) Positive germ tube formation in human serum confirmed the diagnosis of *Candida albicans.* C) Antifungal sensitivity assay on Mueller-Hinton agar for Fluconazole (Flu; 25 μg), Itraconazole (ITC; 8 μg), Nystatin (NY; 100 U), and Amphotericin B (AMB; 10 μg) showed strongest inhibition with Itraconazole.

Culture of the fresh mid-stream urine sample on Sabouraud Dextrose Agar at 37 °C for 24 hours revealed *Candida* sp *.* colonies. Subculturing, to ensure purity and optimal growth, with chloramphenicol, confirmed the presence of large white, round, curved, soft and smooth to wrinkle colonies with a characteristic yeast odor ( Figure 1A). Examination of air-dried fixed smears stained with Gram's stain under a light microscope using 100× oil immersion objective showed gram positive budding yeast cells indicative of *Candida albicans* that was confirmed by positive germ tube formation by germ tube test ( Figure 1B). Briefly, one colony was emulsified in human serum and incubated at 37 °C for two hours. Five or more germ tubes (short and aseptate germinating hyphae)/HPF were observed in a wet mount preparation. Disk diffusion antifungal susceptibility testing was conducted using *C. albicans* ATCC 90028 as a standard control strain. An inoculum from a 48-hour old culture with ~1 – 5 × 10 ^6^ CFU/mL was plated on the dry surface of Mueller-Hinton agar supplemented with 2% glucose and 0.5 μg/mL methylene blue. After 3–5 minutes, the antifungal disks for Fluconazole (25 μg), Itraconazole (8 μg), Nystatin (100 U), and Amphotericin B (10 μg) were applied with gentle pressure (Rev.7/03.12.2012; Liofilchem ^®^ S.r.l., Italy). After 24 hours of aerobic incubation at 35 °C, the diameter of the growth inhibition zone was manually measured for comparison of the antifungal sensitivity that turned highest with Itraconazole followed by Nystatin, Amphotericin B then the least effective was Fluconazole ( Figure 1C).

Insulin Glargine 40 IU SC daily with Insulin Aspart SC six hourly was administered with dose adjustment according to sliding scale, along with normal saline (0.9%) I/V infused. I/V antifungal, Amphotericin B (Fungizone), 40 mg/day for five days as a five hour I/V infusion was administered.

At the end of the five day treatment period, these interventions led to rapid improvement in the short-term glycemic control marker and the patient regained his consciousness, was cooperative and oriented to time and place. Furthermore, his urine became normal, free from the yeast and no more ethanol was detectable in the urine or blood.

## Discussion

Traditional auto-brewery syndrome is linked to gut fermentation but urinary auto-brewery cases have been rarely reported. ^14, 15^ The latter author reported a postmortem case report. The drunk legal limits of 30–80 mg/dL blood ethanol level vary from country to country and from one profession to another. Endogenous sources of ethanol are gut fermentation and intermediary metabolism, and is cleared by hepatic class I alcoholic dehydrogenase, with a K _m_ value of 5–10 mg, where the healthy liver disposes of 0.1 g/kg body weight/hour. ^16^ Reportedly, physiological levels of blood ethanol in healthy controls have a range of 0.0–39 mg/L. ^8^ In Saudi Arabia, a population-based study was done to measure endogenous blood ethanol level in a representative sample of 1400 abstinent residents using the sensitive headspace gas chromatography/mass spectrophotometry. Results showed a mean ± SD (and range) of 0.14 ± 0.35 (0.00–1.53) mg/dL. ^17^

The incidence of candidiasis has increased in number over the years and is linked to significant morbidity and mortality in critically ill and immunosuppressed patients. DM is a major risk factor for candiduria. Diabetic patients have an increased propensity to *Candida* sp *.* infections due to disease-related immunosuppression, enriching glycosuria and various other physiological alterations. ^10- 13^ The rate of candiduria is 10% among T2DM patients, where *Candida albicans* constituted ~50% of the isolates and non-albicans *Candida* were more linked to symptomatic candiduria. Nearly, 80% of these T2DM patients had hemoglobin A1c >7%. There were strong positive correlations between candiduria and each of urine acidity (being acidic; 5–6 pH), glycosuria (≥3 pluses) and HbA1c % (>7%); all of them help colonization. ^18^ Indeed, the prevalence of candiduria among T2DM patients varies greatly in the previously published studies, including Middle Eastern ones, and showed a range of 2.7–30%. ^11, 19- 22^

The present case was fortunate, as 1) the patient’s liver and kidney functions seemed normal, 2) his long-term glycemic control was fair as HbA1c ≤7.6%, and 3) there is no appreciable Gaptin-ethanol drug interaction. On the contrary, Gaptin may lessen the effects of alcohol intoxication and dependency. ^23^

Despite the notion that diabetes is a risk factor for candiduria, only two case reports ^14, 15^ connected the ability of *Candida* to produce ethanol in vivo and in vitro from the large amount of glucose escaping into the urine in poorly controlled diabetes. However, they did not appreciate the possibility of alcohol absorption through the urinary bladder and urethral epithelium, reporting undetectable blood ethanol levels. The concentration gradient is the only driving force for the free movement of the small polar uncharged ethanol molecules through the cellular membranes and among body compartments. Such alcohol absorption has long been reported to happen during the bladder irrigation for the transurethral resection and vaporization of the prostate using ethanol-containing irrigation fluid; even to an intoxicating level. ^24- 31^ Opposite to the gut, venous drainage of the urinary bladder joins general circulation before reaching the liver. ^32^ Therefore, absorbed ethanol circulates to the central nervous system before the subsequent hepatic clearance.

Levels of endogenous ethanol reaching 300 mg/dL in blood and 600 mg/dL in urine was also reported in abstainer T2DM Pakistani patients with random blood sugar of >250 mg/dL and disease duration of more than five years. Although the investigators did not check its presence, they suggested candiduria as the source of ethanol along with the hyperglycemic ketotic intermediary metabolism. Such endogenous ethanol could be a complicating factor for the known neuropathic complication of diabetes. ^33^ Similar results were reported by Liebich's group ^34- 38^ investigating ethanol production and excretion in both type 1 DM (T1DM) and T2DM. They reported that T1DM but not T2DM patients excreted significantly more ethanol in urine than healthy control subjects. However, the rate of urinary alcohol did not relate to diabetic peripheral neuropathy in both groups. In their earlier study, increased urinary alcohol was recorded for both T1DM and T2DM (on hypoglycemic drugs or diet control), although it was more significant for those on insulin.

The presentation and investigations of this case point to urinary ethanol as a major source. Nevertheless, a gut source of blood ethanol cannot be excluded in light of the patient’s reported frequent constipation. Moreover, incompliance with the antipsychotic Gaptin could have participated in the observed intoxication-like psychiatric and awareness deterioration in the week of chaos.

## Conclusion

The reporting of this elderly case of urinary auto-brewery insulin-dependent T2DM was to alert physicians to an overlooked and a rarely encountered presentation of a poorly controlled case of diabetes. Ethanol endogenously produced by fermentation in the urinary system, and other sources, could bring more chaos to the scenario. A future large survey study could use sensitive breathe alcohol analyzers to scan a large number of diabetic patients followed by correlation with potential candiduria and prognostic factors. Ethanol absorption through the bladder and urethral walls needs further assiduous dissection.

## Data availability

All data underlying the results are available as part of the article and no additional source data are required.
