# Supplementary material for: Case Report: Diabetic urinary auto-brewery and review of literature
Source: F1000Res. 2021 May 20;10:407. [Version 1] doi: 10.12688/f1000research.52461.1 (PMC8886172; doi:10.12688/f1000research.52461.1)
Supplement: Peer review history

# Peer review: Reviewer response for version 1

**DOI:** 10.5256/f1000research.55742.r122175

**Published:** 2022-02-28

**Authors:** Abdel-Naser Elzouki

**Affiliations:** 1 Internal Medicine, Hamad Medical Corporation, Doha, Qatar

The author is reporting a rare case of elderly patient known to have insulin-dependent type 2 diabetes with urinary auto-brewery syndrome treated effectively with antifungal therapy.

 The report is well written and the figures are clear and supporting the findings and diagnosis.  

 This case is of important clinical impact and open the line for future studies in large cohort of diabetic patients to study in details this association.

Are enough details provided of any physical examination and diagnostic tests, treatment given and outcomes?

Yes

Is the case presented with sufficient detail to be useful for other practitioners?

Yes

Is sufficient discussion included of the importance of the findings and their relevance to future understanding of disease processes, diagnosis or treatment?

Yes

Is the background of the case’s history and progression described in sufficient detail?

Yes

Reviewer Expertise:

Metabolic diseases including diabetes, NAFLD, NASH and related conditions in the field of diabetes and liver diseases. Also in anticoagulation and VTE.

I confirm that I have read this submission and believe that I have an appropriate level of expertise to confirm that it is of an acceptable scientific standard.

# Peer review: Reviewer response for version 1

**DOI:** 10.5256/f1000research.55742.r85773

**Published:** 2021-06-09

**Authors:** Tarek Abdulraoof Salem

**Affiliations:** 1 Department of Medical Biochemistry, College of Medicine, Qassim University, Buraidah, Saudi Arabia

The submitted report describes the production of endogenous ethanol by fermentation in the urinary system of an 85-year-old uncontrolled diabetic patient. Auto-brewery syndrome, in the setting of little alcohol consumption or teetotalers, is rarely reported probably because of under diagnosis. High endogenous ethanol production and blood levels, due mainly to a sedentary lifestyle with a high carbohydrate diet and enhanced gut fermentation has been reported. This report is to alert physicians to an overlooked and a rarely encountered presentation of a poorly controlled case of diabetes.

 It is well-constructed and has good originality. The author covered all clinical and laboratory aspects of the case in a good manner. Most of details related to the case were mentioned and discussed. The only concern is how the author cooperated with the Egyptian team to get the details of that case?

 I recommend accepting this case report for publishing in the journal.

Are enough details provided of any physical examination and diagnostic tests, treatment given and outcomes?

Yes

Is the case presented with sufficient detail to be useful for other practitioners?

Yes

Is sufficient discussion included of the importance of the findings and their relevance to future understanding of disease processes, diagnosis or treatment?

Yes

Is the background of the case’s history and progression described in sufficient detail?

Yes

Reviewer Expertise:

Endocrinology and immunology

I confirm that I have read this submission and believe that I have an appropriate level of expertise to confirm that it is of an acceptable scientific standard.
